# Cordycepin activates AMP-activated protein kinase (AMPK) *via* interaction with the γ1 subunit

**DOI:** 10.1111/jcmm.12187

**Published:** 2013-11-28

**Authors:** Chongming Wu, Yanshen Guo, Yan Su, Xue Zhang, Hong Luan, Xiaopo Zhang, Huixin Zhu, Huixia He, Xiaoliang Wang, Guibo Sun, Xiaobo Sun, Peng Guo, Ping Zhu

**Affiliations:** a Pharmacology and Toxicology Research Center, Institute of Medicinal Plant Development, Chinese Academy of Medical Sciences, Peking Union Medical CollegeBeijing, China; b State Key Laboratory of Bioactive Substance and Function of Natural Medicines & Ministry of Health Key Laboratory of Biosynthesis of Natural Products, Institute of Materia Medica, Chinese Academy of Medical Sciences and Peking Union Medical CollegeBeijing, China

**Keywords:** Cordycepin, AMPK, LKB1, Molecular docking

## Abstract

Cordycepin is a bioactive component of the fungus *Cordyceps militaris*. Previously, we showed that cordycepin can alleviate hyperlipidemia through enhancing the phosphorylation of AMP-activated protein kinase (AMPK), but the mechanism of this stimulation is unknown. Here, we investigated the potential mechanisms of cordycepin-induced AMPK activation in HepG2 cells. Treatment with cordycepin largely reduced oleic acid (OA)-elicited intracellular lipid accumulation and increased AMPK activity in a dose-dependent manner. Cordycepin-induced AMPK activation was not accompanied by changes in either the intracellular levels of AMP or the AMP/ATP ratio, nor was it influenced by calmodulin-dependent protein kinase kinase (CaMKK) inhibition; however, this activation was significantly suppressed by liver kinase B1 (LKB1) knockdown. Molecular docking, fluorescent and circular dichroism measurements showed that cordycepin interacted with the γ1 subunit of AMPK. Knockdown of AMPKγ1 by siRNA substantially abolished the effects of cordycepin on AMPK activation and lipid regulation. The modulating effects of cordycepin on the mRNA levels of key lipid regulatory genes were also largely reversed when AMPKγ1 expression was inhibited. Together, these data suggest that cordycepin may inhibit intracellular lipid accumulation through activation of AMPK *via* interaction with the γ1 subunit.

## Introduction

AMP-activated protein kinase (AMPK) is a key cellular energy sensor that plays a critical role in the regulation of lipid metabolism 1. Upon activation, AMPK phosphorylates and inhibits enzymes involved in fatty acid biosynthesis such as acetyl-CoA carboxylase (ACC), fatty acid synthase (FAS) and HMG-CoA reductase (HMGR) 2,3. AMP-activated protein kinase also regulates the transcription of genes involved in lipid metabolism, such as peroxisome proliferator–activated receptor α (PPARα) 4, PPARγ 5 and sterol regulatory element–binding protein 1c (SREBP1c) 6. An increasing body of evidence has shown that many metabolic disorders are commonly associated with the dysregulation of AMPK 7. AMP-activated protein kinase has been proposed to be a major therapeutic target for the treatment of obesity and obesity-linked metabolic disorders such as hyperlipidemia and fatty liver 8.

AMP-activated protein kinase can be activated through several different mechanisms, the most common being the binding of AMP to the γ subunit of AMPK, resulting in conformational changes and thus exposing the active site (Thr-172) on the α subunit 9. AMP-activated protein kinase is a heterotrimeric enzyme composed of catalytic (α1/α2), scaffold (β1/β2) and regulatory (γ1/γ2/γ3) subunits. Of the three AMPKγ isoforms, the γ1 isoform is the major regulatory subunit accounting for more than 80% of total AMPK activity in most tissues. The γ2 isoform contributes about 20% of total AMPK activity while the γ3 isoform for a minimum contribution 10. Another way to activate AMPK involves phosphorylation of AMPK at the Thr-172 on the α subunit by AMPK kinases such as liver kinase B1 (LKB1) and calmodulin-dependent protein kinase kinase (CaMKK) 1. A number of pharmacological agents, including metformin 11, thiazolidinediones 12 and natural products 13,14, have been shown to activate AMPK both *in vitro* and *in vivo*. Although the underlying mechanisms by which these agents activate AMPK are not fully understood, the data indicate that they act in an indirect manner. In contrast, the compounds A-769662 15 and PT1 16 can activate AMPK by binding directly to either the γ or α subunits.

Cordycepin, also known as 3′-deoxyadenosine, is a bioactive component of the fungus *Cordyceps militaris*. It has been shown to possess multiple pharmacological activities such as inhibition of tumour growth, modulation of the immune response and suppression of reactive oxygen species 17. Recently, we and other groups have reported that cordycepin can potently reduce lipid levels in high fat diet–induced hyperlipidemic animals 17,18. Further studies showed that the lipid-decreasing effects of cordycepin may be caused by the activation of AMPK 18. Hyperlipidemia is an important risk factor for many life-threatening diseases, predominantly, cardiovascular diseases (CVD) 19,20. The lipid-lowering effect of cordycepin proposed a potential use of it in the treatment of hyperlipidemia and hyperlipidemia-related disorders such as diabetes and atherosclerosis. However, the precise mechanisms of cordycepin's effects on lipid regulation and AMPK activation are still unknown. Concerning these issues, the lipid-lowering and AMPK-activating effects of cordycepin were investigated in HepG2 cells in this study to provide insight into the mechanisms of cordycepin on regulating lipid metabolism and activating AMPK.

## Materials and methods

### Materials

HepG2 cells, which originated from the American Type Culture Collection (ATCC; Manassas, VA, USA), were obtained from the Peking Union Medical College. Ni^2+^-nitrilotriacetic acid (NTA) agarose was purchased from General Electric (Fairfield, CT, USA). Anti-AMPKα1(#2795), anti-AMPKγ1(#4187), anti-ACC(#3662), anti-pAMPK (Thr-172) (#2535), anti-pACC (S79) (#3661) and anti-β-Actin (#8456) were purchased from Cell Signaling Technology (Beverly, MA, USA). 5-Aminoimidazole-4-carboxyamide ribonucleoside (AICAR), compound C, STO-609, 8-CPT and MRS1191 were purchased from Sigma-Aldrich Co. Ltd. (St. Louis, MO, USA). Cordycepin, with a purity of 99% as determined by high-performance liquid chromatography (HPLC), was prepared by our group as previously reported 21.

### Cell culture and transfection

Cells were maintained in DMEM (Gibco-BRL, Grand Island, NY, USA) supplemented with 10% FBS and grown to 70–80% confluence, then incubated in 0.02% bovine serum albumin (BSA; Sigma-Aldrich) in DMEM for 24 hrs. Cells were then washed and incubated with the indicated concentration of either the respective chemicals in 0.02% BSA/DMEM or 0.02% BSA/DMEM alone for the indicated periods of time. Plasmid DNA or siRNAs were transfected into HepG2 cells using Lipofectamine 2000 (Invitrogen, Carlsbad, CA, USA) according to manufacturer's instruction after cells were seeded for 12 hrs.

### Lipid droplet size measurement

HepG2 cells were transfected with lipid droplet (LD) specific green fluorescent protein (GFP)-perilipin An expression vector then treated with oleic acid (OA, 100 μM) with or without cordycepin (1 μM) for 16 hrs. After fixed in paraformaldehyde for 1 hr, images for GFP-positive hepatocytes were acquired and the diameter of the largest LD in each cell was measured by AxioVision (Carl Zeiss, Jena, Germany). About 100 cells for each condition were counted for statistical analysis.

### Western blotting

HepG2 cells were lysed in lysis buffer containing 10% glycerol, 1% Triton X-100, 135 mM NaCl, 20 mM Tris (pH 8.0), 2.7 mM KCl, 1 mM MgCl_2_, and protease and phosphatase inhibitors (0.5 mM PMSF, 2 μM pepstatin and 2 μM okadaic acid). Aliquots of samples were subjected to SDS-PAGE followed by transfer to polyvinylidene difluoride (PVDF) membranes (Amersham Pharmacia, Uppsala, Sweden). Immunoblotting was performed with respective antibodies (1:1000). Following incubation with horseradish peroxidase–conjugated secondary antibody (Sigma-Aldrich, Shanghai, China), proteins were detected with ECL plus kits (Amersham, Piscataway, NJ, USA).

### ULight™-Acetyl-CoA Carboxylase (Ser79) Peptide (SAMS peptide) assay

AMP-activated protein kinase protein activity was assessed under typical assay conditions in a 50-μl reaction mixture containing 20 mM Tris-HCl (pH 7.5), 1 mM dithiothreitol, 5 mM MgCl_2_, 50 mM NaCl, 50 μM ATP (0.4 μCi of [γ-176 33P] ATP per reaction) and 50 μM SAMS peptide. The reaction was initiated by the addition of AMPK protein (100 nM), incubated at 30°C for 10 min., and terminated by the addition of 50 μl of 1% H_3_PO_4_. The particulate matter was then transferred to P30 filter paper and washed three times with 0.1% H_3_PO_4_. Radioactivity that had been incorporated into AMPK was determined by liquid scintillation counting with a Wallac Microbeta plate counter. Background radioactivity was quantified from reactions without enzyme and subtracted from all of the samples.

### Lipid synthesis and lipid oxidation assays

The rate of *de novo* fatty acid synthesis was determined based on the rate of [1-^14^C]acetate incorporation into fatty acids during a 2-hr period, as previously described 22. Fatty acid oxidation was measured in cells that were incubated for 24 hrs with the indicated compounds. The [1-^14^C]palmitate (0.2 mCi/ml) assay was performed based on the methods of Tavridou *et al*. 23.

### HPLC

HepG2 cells were incubated with cordycepin (0.1–10 μM) or equal volume of DMSO for 1 hr, then quickly harvested into phosphate-buffered saline and immediately centrifuged for 2 min. at 1000 × *g* (4°C). The pellets were resuspended in 150 μl of perchloric acid (4% v/v) and incubated on ice for 30 min. Within 1 hr, the lysates were adjusted to pH 6–8 with 2 M KOH/0.3 M MOPS and incubated for 30 min. on ice. A precipitated salt was separated from the liquid phase by centrifugation at 13,000 × *g* for 10 min. The samples were aliquoted and stored at −80°C until further analysis. Adenine nucleotide quantification was assessed using a Waters ACQUITY UPLC system (Waters, Milford, MA, USA) with a UPLC HSS T3 C18 column (2.1 × 150 mm, 1.8-μm particle size; Waters). Chromatographic separation was performed with a gradient of mobile phases A (20 mM ammonium acetate, adjusted to pH 4.0 with acetic acid) and B (acetonitrile). The flow rate of the mobile phase was 0.25 ml/min. The gradient programme was as follows: 0–3 min. with 98% of A, 3–8 min. from 98% to 88% of A, 8–16 min. with 88% of A, 16–17 min. from 88% to 98% A, and 98% of A for 17–21 min. to equilibrate the column prior to the next injection.

### Molecular docking by FlexX program

The PDB file of AMPK (No. 2Y94) was selected from PDB bank and the docking process was performed by FlexX program. FlexX is a fast flexible automated docking program that considers ligand conformational flexibility by an incremental fragment placing technique. The initial structure of cordycepin was constructed by SYBYL 7.2 and the geometry was subsequently optimized using the TRIPOS force field, Gasteiger–Huckel charges and Powell method; a non-bond cut-off of 8 Å was adopted to consider the intramolecular interaction. For investigating the interaction of cordycepin with various AMPK subunits, the FlexX program interfaced with SYBYL7.2 was used to dock cordycepin to each subunit of AMPK.

### Cloning, expression and purification of the AMPKγ1 subunit

The AMPKγ1 gene was obtained from HepG2 cells by RNA extraction and was then reverse-transcribed. The cDNA sequence was amplified by PCR using the primers 5′-GGAATTCCATATGAAGTCTCATCGCTGCTATGAC-3′ and 5′-CGGGATCCTCAGGGCTTCTTCTCTCCACCTG-3′. The expression vector of AMPK was constructed with pET21d and transformed into the competent *Escherichia coli* strain BL21 (DE3). The fusion proteins were purified from a clarified bacterial lysate by Ni^2+^-affinity chromatography and analysed by SDS-PAGE.

### Fluorescent measurements

The binding of cordycepin to AMPKγ1 was first assessed by fluorescence quenching method. His-tagged AMPKγ1 was dissolved in 200 μl of PBS buffer (10.0 μM, pH 7.4) to a final concentration of 2.0 μM. Various amounts of cordycepin were added into the AMPKγ solution making the resultant ratios of protein *versus* drugs ranging from 1:1 to 1:4. The fluorescence intensities were recorded using a Tecan Infinite M1000Pro Microplate Reader (TECAN Group Ltd, Shanghai, China) with exciting wavelength at 230 nm and recording emission spectra in 290–450 nm. The static quenching constant of cordycepin to AMPK γ1 was calculated by Stern–Volmer equation as previous reported 24. All tests were repeated in triplicate.

### Circular dichroism measurements

Circular dichroism (CD) measurements were performed on a JASCO-810 spectropolarimeter (Tokyo, Japan). Fusion proteins both with and without cordycepin were made in the range of 200–250 nm using a 0.5-cm cell at 0.2-nm intervals with three scans averaged for each CD spectra. The concentration of AMPKγ1 protein was fixed at 2.7 μM in 10.0 μM PBS buffer with pH 7.4, and the molar ratios of protein to cordycepin ranged from 1:1 to 1:8.

### Generation of AMPKγ1 stable knockdown cell line by lentivirus

A DNA fragment encoding an siRNA specific for AMPKγ1 (5′-CCGGGCTAGAAGAACACAAGATATTCAAGAGATATCTTGTGTTCTTCTAGCTTTTTTG-3′) was inserted into the FG12 expression vector and packaged into lentivirus as previously described 25. Lentivirus packaging and stable cell line generation were performed as previously described 26. HepG2 cells were infected for 12 hrs with the lentivirus expressing the AMPKγ1-specific siRNA. After six passages, infected cells that stably expressed the siRNA were used as an AMPKγ1 knockdown cell line. The knockdown efficiency was confirmed by both quantitative real-time PCR and western blot. A lentivirus generated from the empty vector was used as the siNC control.

### Real-time quantitative PCR

The mRNA levels of lipid metabolism-related genes were determined by real-time quantitative PCR. Total RNA extraction, cDNA synthesis and quantitative PCR assays were performed as described previously 27. At least three independent biological replicates were performed to check the reproducibility of the data. The gene-specific primers used for quantitative PCR are listed in Table S1.

Analysis of other methods such as the 3-(4,5-Dimethylthiazol-2-yl)-2,5-diphenyltetrazolium bromide (MTT) assay and oil-red O staining were performed routinely as previously reported 18,28.

### Statistical analysis

Data are presented as the mean ± SEM. One-way anova was used to determine significant differences among groups, after which the modified Student's *t*-test with the Bonferroni correction was used for comparison between individual groups. *P* < 0.05 was considered statistically significant. All statistical analyses were performed with SPSS 13.0 software (SPSS Inc., Chicago, IL, USA).

## Results

### Cordycepin inhibits OA-elicited lipids accumulation, alters lipid biosynthesis and oxidation rates, and decreases the size of LDs in HepG2 cells

**Fig 1:**
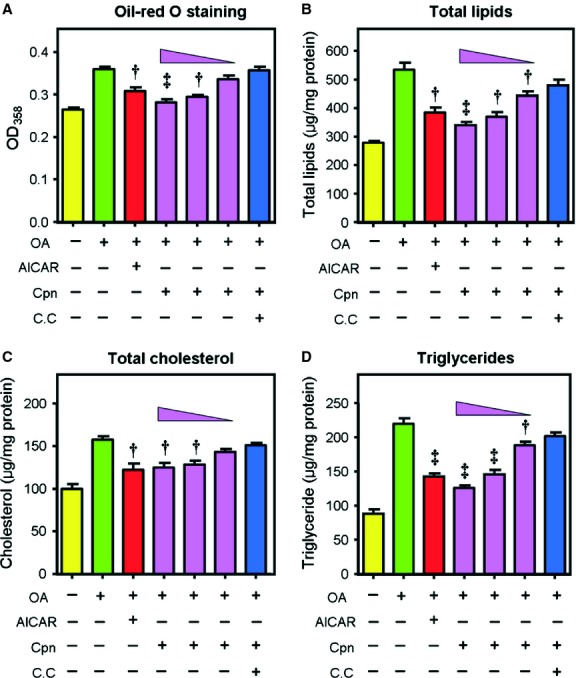
Cordycepin inhibits intracellular lipid accumulation in HepG2 cells. Cells were incubated with oleic acid (OA, 100 μM) for 12 hrs, then treated with various agents [5-Aminoimidazole-4-carboxyamide ribonucleoside (AICAR) (1 mM), cordycepin (0.1–10 μM) or cordycepin + compound C (10 μM + 40 μM)] for additional 6 hrs. Neutral lipids were determined by spectrophotometry at 358 nm after oil-red O staining (A); total lipids (B), total cholesterol (C) and triglycerides (D) were measured by kits according to the manufacturer's instructions. Bars depict the means ± SEM of at least three experiments. Asterisks represent statistically significant differences from the OA-overloaded control group (^†^*P* < 0.05, ^‡^*P* < 0.01). cpn: cordycepin; C.C; compound C.

**Fig 2:**
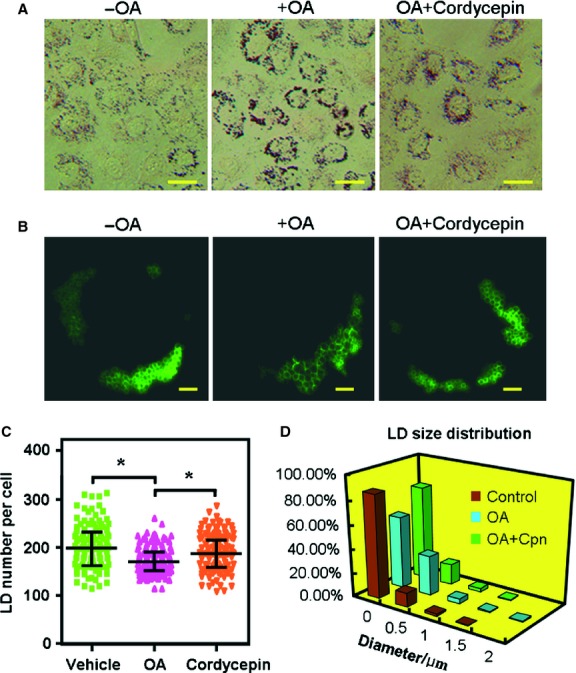
Cordycepin decreases LD size in HepG2 cells. Cells were transfected with the LD-specific GFP-Perilipin A expression vector followed by treatment with OA (OA, 100 μM) either with or without cordycepin (1 μM) for 16 hrs. The LDs were visualized by oil-red O staining (bar = 50 μm; A) and fluorescence microscopy (bar = 5 μm; B). Over 100 fluorescence photos were taken for each group, and the LD number (C) and the diameter of the largest LD (D) in each cell were recorded and plotted. Asterisks represent statistically significant differences from the OA-treated control group (*P < 0.05). OA: oleic acid; LD: lipid droplet.

**Fig 3:**
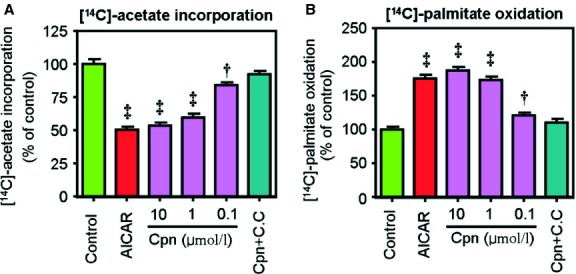
Cordycepin inhibits *de novo* lipogenesis (A) and promotes fatty acid oxidation (B). HepG2 cells were incubated with either [1-^14^C]acetate for 2 hrs or [1-^14^C]palmitate for 24 hrs co-treated with AICAR (1 mM), cordycepin (0.1–10 μM) or cordycepin + compound C (10 μM + 40 μM), and the radioactivity in the saponifiable fatty acid fractions was measured. The data depict the means ± SEM of at least three experiments. Asterisks represent statistically significant differences from the oleic acid–treated control group (^†^*P* < 0.05, ^‡^*P* < 0.01). cpn: cordycepin; C.C; compound C.

In HepG2 cells, treatment with cordycepin (10 μM) significantly decreased the intracellular levels of triglycerides (TG) and total cholesterol (TC; Fig. S1A and B) and these effects were more pronounced in OA-loaded conditions. As shown in Figure 1, supplementation with cordycepin (0.1–10 μM) inhibited the OA-elicited accumulation of total lipids, TC and TG in a dose-dependent manner. The lipid-lowering effects of cordycepin at 1.0 and 10 μM were comparable to 1 mM of AICAR (Fig. 1). These lipid-lowering effects were also observed in muscle cells such as C2C12 microtubes (Fig. S1C and D). MTT assay showed that cordycepin had a minimal effect on the proliferation of HepG2 cells at concentrations up to 10 μM (Fig. S2), and the typical concentration of cordycepin that induces cytotoxicity is above 100 μM 29, suggesting that the cordycepin-induced hypolipidemic effect was not due to the cytotoxicity of cordycepin. Additionally, the size of LDs, which is the primary storage of intracellular TG, was significantly decreased after treatment with cordycepin (Fig. 2). The percentage of LDs with larger diameters was decreased by cordycepin (Fig. 2D). At the same time, the [1-^14^C]acetate incorporation assay and [1-^14^C]palmitate oxidation assay showed that treatment with cordycepin (0.1–10 μM) significantly decreased the lipid biosynthesis rate and increased the lipid oxidation rate in a dose-dependent manner (Fig. 3). However, the effects of cordycepin on intracellular lipid accumulation and on lipid biosynthesis and oxidation were substantially abolished when compound C, a specific AMPK antagonist, was simultaneously added with cordycepin (Figs 1 and 3; Fig. S1A–D).

### Cordycepin activates AMPK in a dose-dependent manner

**Fig 4:**
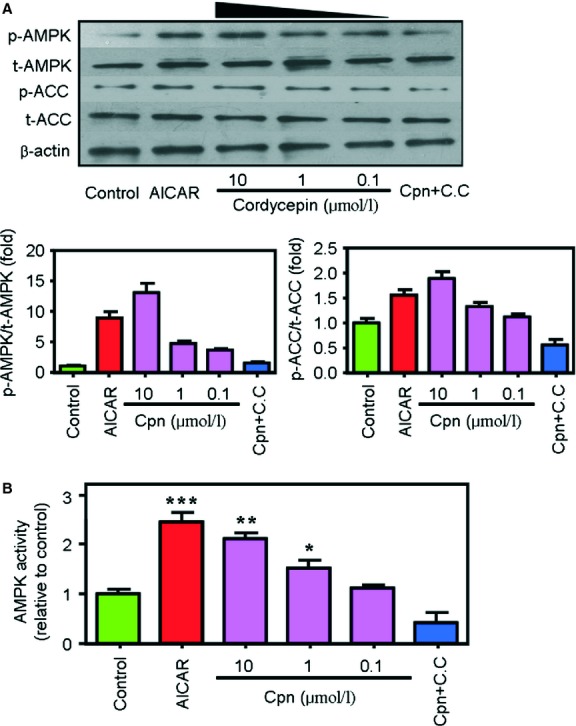
Cordycepin increases AMP-activated protein kinase (AMPK) and acetyl-CoA carboxylase (ACC) phosphorylation (A) and AMPK activity (B). Cells were treated with AICAR (1 mM), cordycepin (0.1–10 μM) or cordycepin + compound C (10 μM + 40 μM) for 1 hr. Phosphorylation of AMPK (pThr172-AMPK) and its substrate ACC (pSer79-ACC) were detected by western blot analysis. The mean grey values of each lane in the western blot photos were quantified by ImageJ 4.1 software and the ratios of p-AMPK/t-AMPK and p-ACC/t-ACC were calculated. The AMPK activity was determined by the SAMS peptide assay. The data depict the means ± SEM of at least three experiments. Asterisks represent statistically significant differences from the oleic acid–treated control group (*P < 0.05, **P < 0.01, **^*^P < 0.001). cpn: cordycepin; C.C; compound C.

Activation of AMPK has been proposed to be a central event in maintaining cellular energy homeostasis and is known to play a key role in fat metabolism 8. As shown in Figure 4A, treatment with cordycepin (0.1–10 μM) significantly increased the levels of phospho-AMPK and phospho-ACC. The ratios of phospho-AMPK/total AMPK and phospho-ACC/total ACC were increased by ∼14.0-and 1.8-fold, respectively, after treatment with 10 μM cordycepin (Fig. 4A). This AMPK-activating effect of cordycepin was further confirmed by SAMS peptide assay, which showed that cordycepin significantly increased AMPK activity at concentrations of 1 and 10 μM (Fig. 4B).

### Activation of AMPK by cordycepin does not involve intracellular AMP changes but is suppressed by LKB1 knockdown

**Fig 5:**
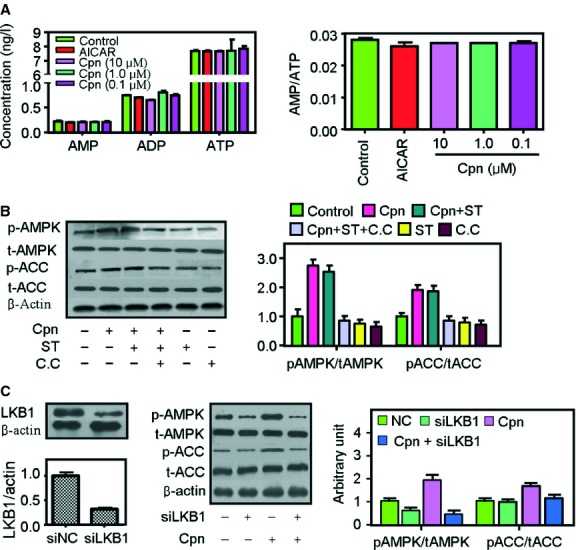
Cordycepin activates AMP-activated protein kinase (AMPK) without changing intracellular AMP level but is inhibited by LKB1 dysfunction. (A) Effect of cordycepin on intracellular AMP, ADP and ATP levels and the AMP/ATP ratio. Cells were treated with AICAR (1 mM), cordycepin (0.1–10 μM) for 1 hr. AMP, ADP and ATP levels were determined by high-performance liquid chromatography (HPLC). (B) Treatment with the CaMKK inhibitor STO-609 (10 μg/ml) did not suppress cordycepin-mediated AMPK and acetyl-CoA carboxylase (ACC) phosphorylation. (C) LKB1 knockdown impaired cordycepin-induced AMPK and ACC phosphorylation. Before western blot analysis, cells were treated with respective agents for 1 hr. The mean grey values of each lane in the western blot photos were quantified by ImageJ 4.1 software and the ratios of p-AMPK/t-AMPK and p-ACC/t-ACC were calculated. The data depict the means ± SEM of at least three experiments. cpn: cordycepin; C.C; compound C; ST: STO609.

Increasing the intracellular AMP/ATP ratio and stimulation of upstream kinases such as LKB1 and CaMKK are the two primary activators of AMPK 1,30. As shown in Figure 5A, neither intracellular concentrations of AMP, ADP and ATP nor the AMP/ATP ratio was significantly changed after treatment with 0.1–10 μM cordycepin. Supplementation with 10 μg/ml of STO-609, a selective CaMKK inhibitor, did not inhibit cordycepin-induced AMPK activation either (Fig. 5B), suggesting that cordycepin can activate AMPK in the absence of CaMKK. In contrast, when the expression of LKB1 was decreased by specific siRNAs (Fig. 5C, left panel), the stimulatory effect of cordycepin on AMPK was dampened (Fig. 5C, right panel). Similarly, the AMPK-stimulating effect of AICAR was also largely impaired in LKB1 knockdown cells (Fig. S3), which was in accordance with previous reports 31 and certified the validity of our results. However, treatment with cordycepin did not increase the phosphorylation of LKB1 (data not shown), implying that cordycepin may act downstream of LKB1.

### Cordycepin interacts with AMPK γ1 subunit *in vitro*

**Fig 6:**
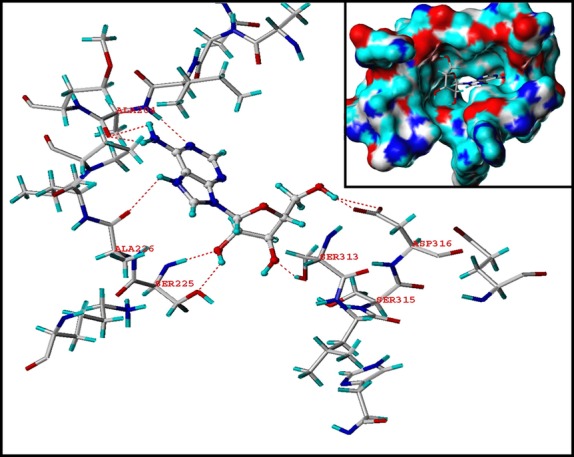
Molecular docking study of the binding affinity of cordycepin to the AMPKγ1 subunit. Hydrogen bonds are represented as dotted lines.

Next, we investigated the interaction between cordycepin and AMPK. The binding ability of cordycepin to each subunit of AMPK was firstly evaluated by molecular docking using FlexX. Cordycepin cannot bind steadily to the α1, α2, β1 or β2 subunits of AMPK (data not shown) but binds to AMPK γ1 subunit with relative high affinity. As displayed in Figure 6, cordycepin is located in the centre of the binding pocket of AMPK γ1 subunit. Hydrophobic interaction and hydrogen bond were the stabilizing force in the interaction. The residues Ala-204, Ala-226, Ser-225, Ser-313 and Asp-316 are key residues corresponding for the binding between AMPKγ1 and cordycepin.

**Fig 7:**
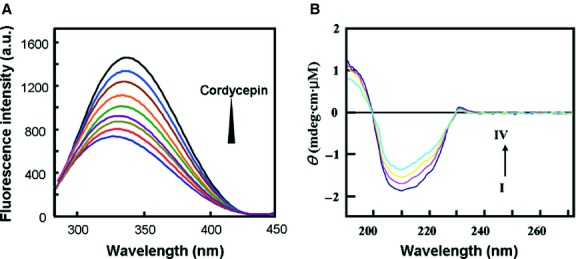
Cordycepin interacts with AMPKγ1 *in vitro*. (A) The fluorescence quenching spectra of AMPKγ1 with different concentrations of cordycepin at 298 K. The concentration of AMPKγ1 was fixed at 2.0 μM, and the ratios of protein *versus* cordycepin ranged from 1:1 to 1:4. (B) The CD spectra of the AMPKγ1-cordycepin system obtained in 10 μM PBS buffer at pH 7.4 at room temperature. The AMPKγ1 concentration was fixed at 2.7 μM, and the molar ratios of protein to cordycepin ranged from 1:1 to 1:8.

The binding of cordycepin with the AMPK γ1 subunit was then assessed *in vitro* by fluorescence and CD spectroscopy. When excited at 230 nm, AMPKγ1 emitted maximal fluorescence at 336 nm. This fluorescence intensity gradually declined with increasing titration of cordycepin (Fig. 7A). Calculation by the Stern–Volmer equation showed that the static quenching constant of cordycepin to AMPK γ1 was 0.3838 ± 0.0521 μM. Furthermore, CD spectroscopy was used to monitor the conformational change in the AMPKγ1 upon interaction with cordycepin. The CD spectra of AMPKγ1 exhibited two negative bands in the UV region at 206 and 220 nm. The α-helicity ratio can be calculated according to the work of Kandagal *et al*. 32, which showed that the α-helicity ratio of AMPK decreased from 21.97% to 15.46% after titration with cordycepin. These results indicated that cordycepin may interact with the AMPK γ1 subunit *in vitro*.

### Knockdown of the AMPK γ1 subunit by specific siRNA substantially abolishes the AMPK-activating and lipid-lowering effects of cordycepin

**Fig 8:**
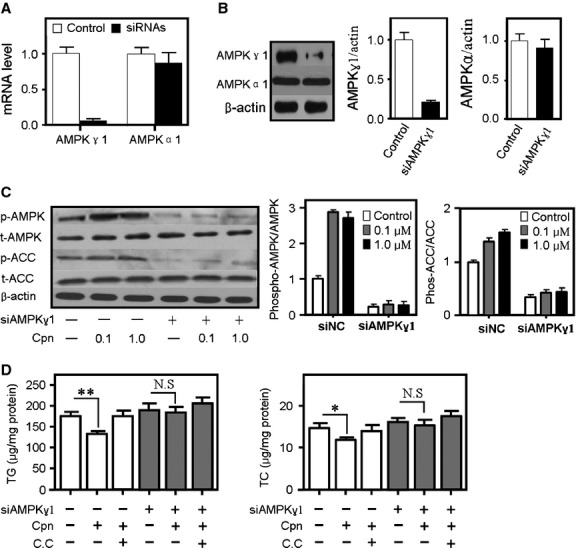
Cordycepin-induced AMP-activated protein kinase (AMPK) phosphorylation was suppressed in HepG2 cells in which AMPKγ1 was stably knocked down. Real-time quantitative PCR (A) and Western blot (B) showed that the expression of AMPKγ1 was significantly decreased, whereas AMPKα1 was not inhibited. Cordycepin-mediated AMPK-and acetyl-CoA carboxylase (ACC)-phosphorylation (C) and TC-and TG-decrease (D) were substantially reduced by knockdown of AMPKγ1. Cells were co-treated with oleic acid (OA, 100 μM) and either cordycepin alone (0.1–10 μM) or cordycepin + compound C (10 μM + 40 μM) for 6 hrs. Total cholesterol (TC) and triglycerides (TG) were measured by kits according to the manufacturers’ protocols. Bars depict the means ± SEM of at least three experiments. cpn: cordycepin; C.C; compound C.

To confirm the key role of AMPK γ1 in cordycepin-induced AMPK activation, four independent stable cell lines in which AMPKγ1 expression was stably suppressed by transduction of a lentiviral construct expressing AMPKγ1-specific siRNA were developed. Interference with AMPKγ1-specific siRNA does not affect the expression levels of AMPKα1 subunit at the either mRNA level or protein level (Fig. 8A and B), nor does it influence the transcription of the other two AMPKγ isoform (AMPKγ2 and AMPKγ3; Fig. S4). However, the stimulating effect of cordycepin on the phosphorylation of AMPK and ACC was dramatically decreased when AMPKγ1 expression was sufficiently inhibited (Fig. 8C). Correspondingly, the effects of cordycepin on the reduction in TC and TG levels were substantially impaired following knockdown of the AMPKγ1 subunit with siRNA (Fig. 8D).

### The regulatory effects of cordycepin on the expression of key lipid metabolic genes were largely reversed in AMPKγ-deficient cells

**Fig 9:**
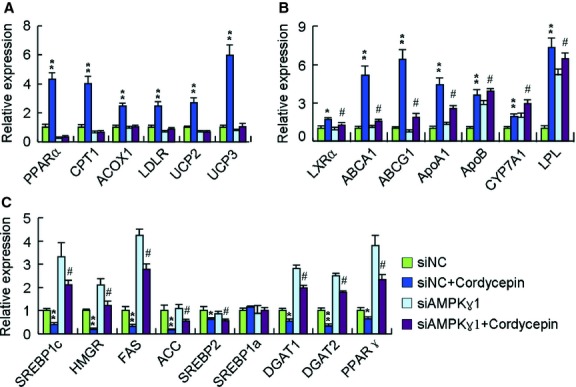
The regulatory effects of cordycepin on the expression of key lipid metabolic genes were reversed in AMPKγ-deficient cells. Cells were treated with oleic acid (OA, 100 μM) and cordycepin (10 μM) for 6 hrs. Real-time PCR was conducted with specific oligonucleotide primers, with the amplification of beta-actin serving as the internal control. The data depict the means ± SEM of at least three experiments. *P < 0.05, **P < 0.01, cordycepin-treated cells *versus* control cells in HepG2 cells transfected with scrambled RNA, ^#^*P* < 0.05, ^##^*P* < 0.01, cordycepin-treated cells *versus* control cells in HepG2 cells transfected with specific AMPKγ1-targeting RNA. ABCA1, ATP-binding cassette transporter A1; ACC, acetyl-CoA carboxylase; ApoA1, apolipoprotein A1; CPT1, carnitine palmitoyltransferase 1; CYP7A1, cytochrome P450 7A1; DGAT, diacylglycerol acyltransferase; FAS, fatty acid synthase; HMGR, HMG-CoA reductase; LDLR, low-density lipoprotein receptor; LPL, lipoprotein lipase; LXRα, liver X receptor α; PPARs, peroxisome proliferator–activated receptors; SREBPs, sterol regulatory element–binding proteins; UCPs, uncoupling proteins.

To further investigate the effect of AMPKγ1 subunit in cordycepin-induced hypolipidemic action, the relative mRNA levels of metabolic lipid genes were determined by real-time quantitative PCR in cordycepin-treated HepG2 cells either with or without AMPKγ1 knockdown. In HepG2 cells in which AMPKγ1 was normally expressed, cordycepin significantly increased the transcription of genes that stimulate lipid consumption and transport such as PPARα, carnitine palmitoyltransferase 1 (CPT1), uncoupling proteins (UCPs), liver X receptor α (LXRα), ATP-binding cassette transporter A1 (ABCA1), ABCG1 and apolipoprotein A1 (ApoA1), *etc*. (Fig. 9A and B), and decreased the mRNA levels of lipogenic genes such as SREBP1c, HMGR, FAS, ACC, SREBP2, diacylglycerol acyltransferase 1 (DGAT1), DGAT2 and PPARγ (Fig. 9C). When the expression of AMPKγ1 was inhibited, the regulatory effects of cordycepin on these genes were substantially reduced, suggesting that cordycepin may regulate the transcription of lipid metabolic genes mainly through an AMPKγ1-mediated pathway.

## Discussion

Cordycepin is a bioactive component of the fungus *C. militaris* showing multiple pharmacological activities. Recently, cordycepin has been demonstrated to prevent hyperlipidemia in animals fed a high fat diet, which were thought to be caused partially by activation of AMPK 18,33. However, the precise mechanisms of cordycepin in lipid regulation and AMPK activation are still unknown.

In this study, the lipid-lowering and AMPK-activating effects of cordycepin were first re-conformed in HepG2 cells. Treatment with cordycepin largely reduced the intracellular levels of total lipids, TC and TG (Fig. 1), which was similar with previous reports 18. Our results further showed that cordycepin simultaneously stimulated lipid oxidation and decreased lipid biosynthesis (Fig. 3) and reduced the size of LD, the primary spot for intracellular lipid deposition (Fig. 2), which reflects the lipid-lowering effect of cordycepin in another aspect. When simultaneously supplemented with compound C, the regulatory effects of cordycepin were substantially diminished (Figs 1 and 3), suggesting that cordycepin may modulate intracellular lipid metabolism through activation of AMPK. Consistent with this hypothesis, cordycepin increased the phosphorylation levels of AMPK and its downstream target ACC in a dose-dependent manner. The SAMS peptide assay also showed that AMPK activity was enhanced by cordycepin (Fig. 4).

Compounds activate AMPK mainly through two pathways: enhancement of both intracellular AMP levels and the AMP:ATP ratio 9, and activation of AMPK upstream kinases, including LKB1 and CaMKK 30,34. In our study, cordycepin changed neither the intracellular levels of AMP, ADP and ATP nor the ratio of AMP/ATP (Fig. 5A), suggesting that treatment with cordycepin does not alter the energy status. Our data also showed that cordycepin-induced AMPK activation was independent of CaMKK, as the selective CaMKK inhibitor STO-609 did not influence the stimulating effect of cordycepin on AMPK (Fig. 5C). However, the AMPK-activating effect of cordycepin was suppressed upon siRNA inhibition of LKB1 expression (Fig. 5B). Western blotting analyses showed that treatment with cordycepin did not increase the phosphorylation of LKB1 (data not shown), which implies that cordycepin may act downstream of LKB1.

As LKB1 can directly phosphorylate AMPK, the speculation that cordycepin may act downstream of LKB1 implied that cordycepin may regulate AMPK activation *via* binding directly to AMPK. AMP-activated protein kinase is a heterotrimeric enzyme composed of catalytic (α1/α2), scaffold (β1/β2) and regulatory (γ1/γ2/γ3) subunits. Of the γ isoforms, the γ1 isoform is the major regulatory subunit, being present in complexes that account for 80–90% of total AMPK activity in all tissues 10. The molecular docking study indicated that cordycepin cannot bind steadily to the α1, α2, β1 or β2 subunits of AMPK (data not shown) but binds to AMPKγ1 with relative high affinity (Fig. 6). Fluorescence and CD spectroscopy analyses confirmed this interaction between cordycepin and AMPKγ1 *in vitro* (Fig. 7). In HepG2 cell line that stably suppressed AMPKγ1 expression by a lentiviral construct expressing AMPKγ1-specific siRNA, the AMPK-activating and lipid-lowering effects of cordycepin were substantially suppressed (Fig. 8), demonstrating that the AMPKγ1 subunit plays a key role in cordycepin-induced AMPK activation. Further studies were performed to distinguish the role of other two γ isoforms in the activation of AMPK by cordycepin. As previously reported 10, AMPKγ3 has only a minimum expression and functional contribution in the liver. We inhibited the expression of AMPKγ2 by specific siRNAs, which showed no significant effect on cordycepin-induced AMPK (Fig. S4B). Therefore, cordycepin may activate AMPK mainly through interaction with the AMPKγ1 subunit. Quantitative real-time PCR also showed that the modulating effect of cordycepin on the transcription of many lipid metabolic genes was substantially reduced when the expression of AMPKγ1 was inhibited (Fig. 9).

As cordycepin is an analogue of adenosine, another speculation exists that cordycepin may exert its actions through activation of adenosine receptors. Indeed, many groups have demonstrated that the anti-cancer effect of cordycepin involved in the activation of A3 receptor but not A1 or A2 receptors 35,36, and the activation of A1 receptors in the heart activates AMPK 37. However, simultaneous treatment with specific inhibitors for adenosine A1 (8-CPT) and A3 (MRS1191) receptors had little impact on cordycepin-mediated AMPK activation (Fig. S5), suggesting that cordycepin exerts AMPK-activating action may be not through adenosine receptors. Takahashi *et al*. also reported that cordycepin can inhibit adipogensis independent of A1, A2 or A3 adenosine receptors 17. These results indicated that the AMPK-activating and lipid-lowering effects of cordycepin may be independent of adenosine receptors.

Overall, we provide evidence that cordycepin plays a significant role in decreasing HepG2 cellular lipid accumulation by increasing AMPK activation. Regulation of or interaction with the AMPKγ1 subunit may be the key mechanism of cordycepin-mediated AMPK activation and lipid reduction. Our results provide new understanding of how natural products such as cordycepin can affect lipid metabolism. If supplementation with cordycepin is found to be as effective at ameliorating hepatic lipid accumulation in humans as was observed in HepG2 cells here and in rodents in our previous study, these findings might provide novel treatment strategies for fatty liver and obesity-related disorders in the future.

## Supporting information

Figure S1 Cordycepin inhibits lipid accumulation in HepG2 and C2C12 cells. (A and B) Cordycepin alone (10 μM) inhibits intracellular triglyceride and total cholesterol contents in HepG2 cells. (C and D) Cordycepin alone (10 μM) inhibits oleic acid (OA)-elicited lipid accumulation in C2C12 myotube cells. (E) Knockdown of AMPKγ1 by specific siRNAs substantially eliminates cordycepin-induced AMPK phosphorylation in C2C12 cells. Bars depict the means ± SEM of at least three experiments. Asterisks represent statistically significant differences from the control group (†P < 0.05, ‡P < 0.01). cpn: cordycepin; C.C; compound C.

Figure S2 Cordycepin shows no signficant toxicity on cell viability in HepG2 cells at 0.01–10 μM. Cells were treated without (control) or with different concentrations of cordycepin (0.01–10 μM) for 24, 48 and 72 hrs respectively. Cell viability was quantified by MTT assay. Results are expressed as percentages of cell numbers relative to DMSO control (as 100+). Data represent the mean ± SEM of four separate experiments.

Figure S3 LKB1 knockdown inhibits AICAR-induced AMPK phosphorylation. LKB1 was transiently knockdown by specific siRNAs. AICAR (1 mM) was added into the medium at 22 hrs after transfection and protein was harvested for western blotting after another 1 hr.

Figure S4 Effect of AMPKγ2 knockdown on cordycepin-induced AMPK phosphorylation. (A) AMPKγ1 knockdown does not affect the expression of other two isoforms of AMPKγ subunit as determined by real-time quantitative PCR. (B) Knockdown of AMPKγ2 does not influence cordycepin-mediated AMPK phosphorylation. Knockdown efficiency was assessed by real-time PCR. cpn: cordycepin.

Figure S5 Inhibitors of adenine receptors A1 (8-CPT, 1 μM) (A) and A3 (MRS1191, 1 μM) (B) do not impair cordycepin (10 μM) -elicited AMPK phosphorylation. Before western blot, cells were treated with indicated reagents for 1 hr. cpn: cordycepin. cpn: cordycepin.

Table S1 Oligonucleotide primers used in this study.
