# Supplementary figures and images for: Cordycepin activates AMP-activated protein kinase (AMPK) via interaction with the γ1 subunit
Source: J Cell Mol Med. 2013 Nov 28;18(2):293–304. doi: 10.1111/jcmm.12187 (PMC3930416; doi:10.1111/jcmm.12187)

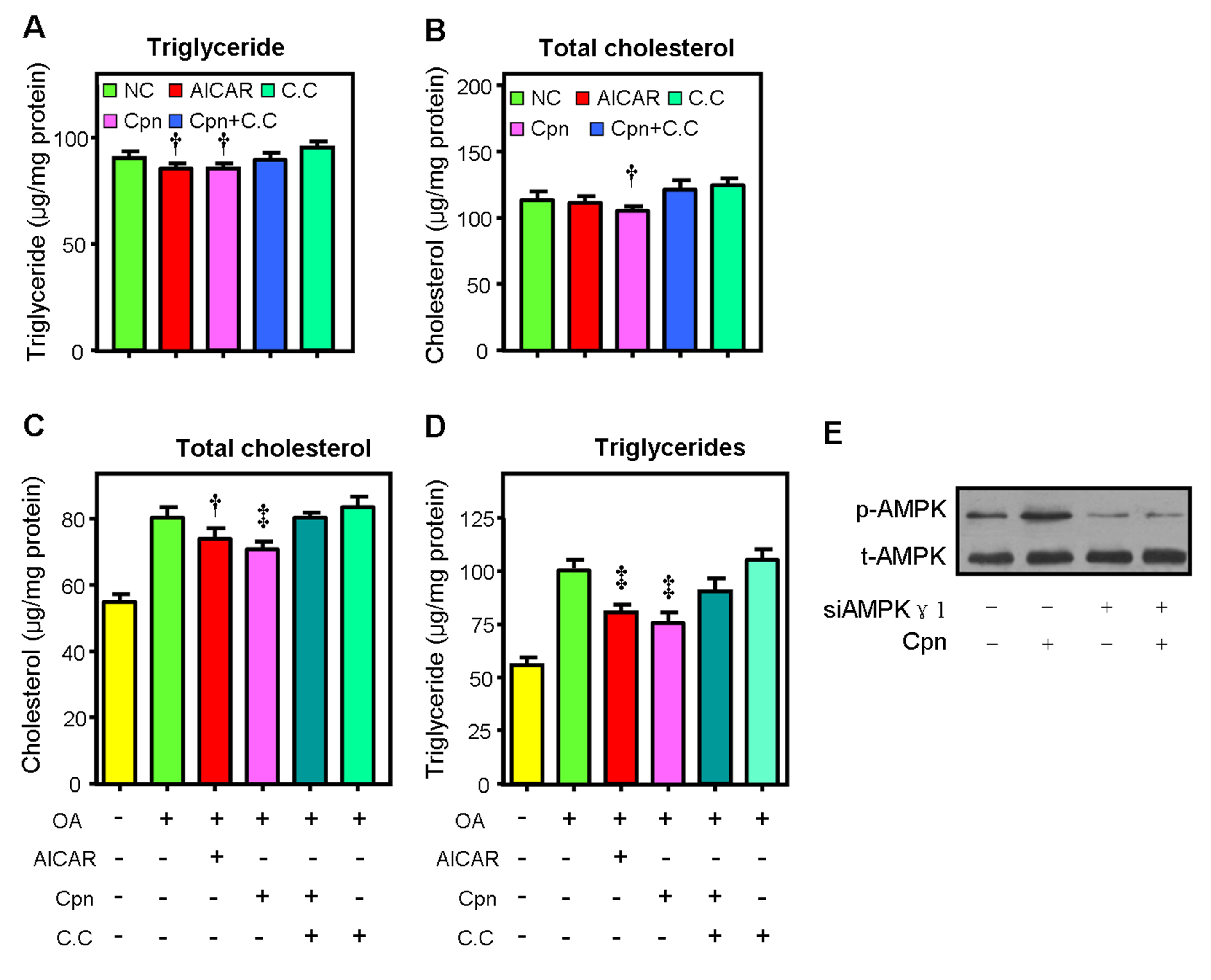

Supplement: Figure S1 — Cordycepin inhibits lipid accumulation in HepG2 and C2C12 cells. (A and B) Cordycepin alone (10 μM) inhibits intracellular triglyceride and total cholesterol contents in HepG2 cells. (C and D) Cordycepin alone (10 μM) inhibits oleic acid (OA)-elicited lipid accumulation in C2C12 myotube cells. (E) Knockdown of AMPKγ1 by specific siRNAs substantially eliminates cordycepin-induced AMPK phosphorylation in C2C12 cells. Bars depict the means ± SEM of at least three experiments. Asterisks represent statistically significant differences from the control group (†P < 0.05, ‡P < 0.01). cpn: cordycepin; C.C; compound C. [file jcmm0018-0293-sd1.tif]

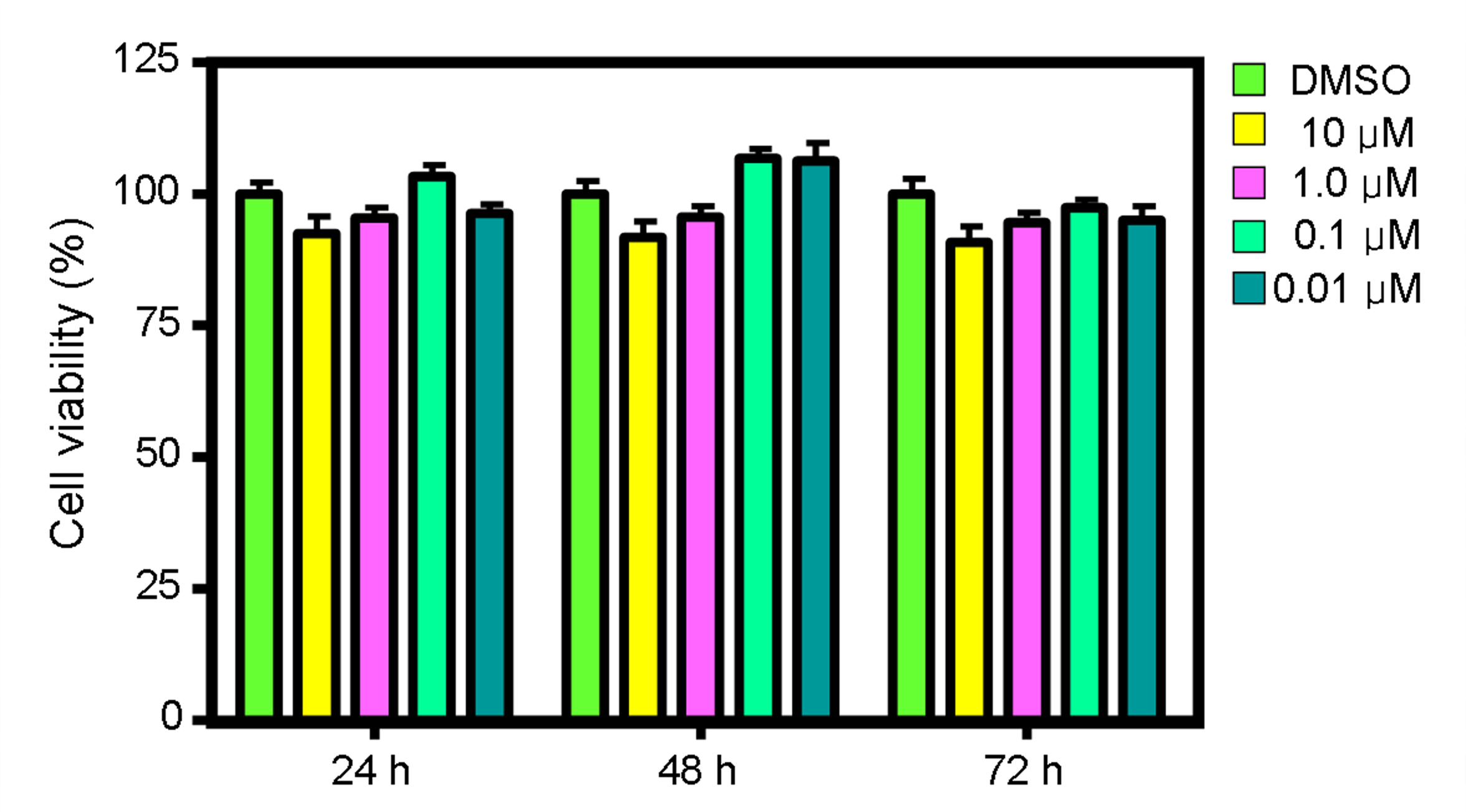

Supplement: Figure S2 — Cordycepin shows no signficant toxicity on cell viability in HepG2 cells at 0.01–10 μM. Cells were treated without (control) or with different concentrations of cordycepin (0.01–10 μM) for 24, 48 and 72 hrs respectively. Cell viability was quantified by MTT assay. Results are expressed as percentages of cell numbers relative to DMSO control (as 100+). Data represent the mean ± SEM of four separate experiments. [file jcmm0018-0293-sd2.tif]

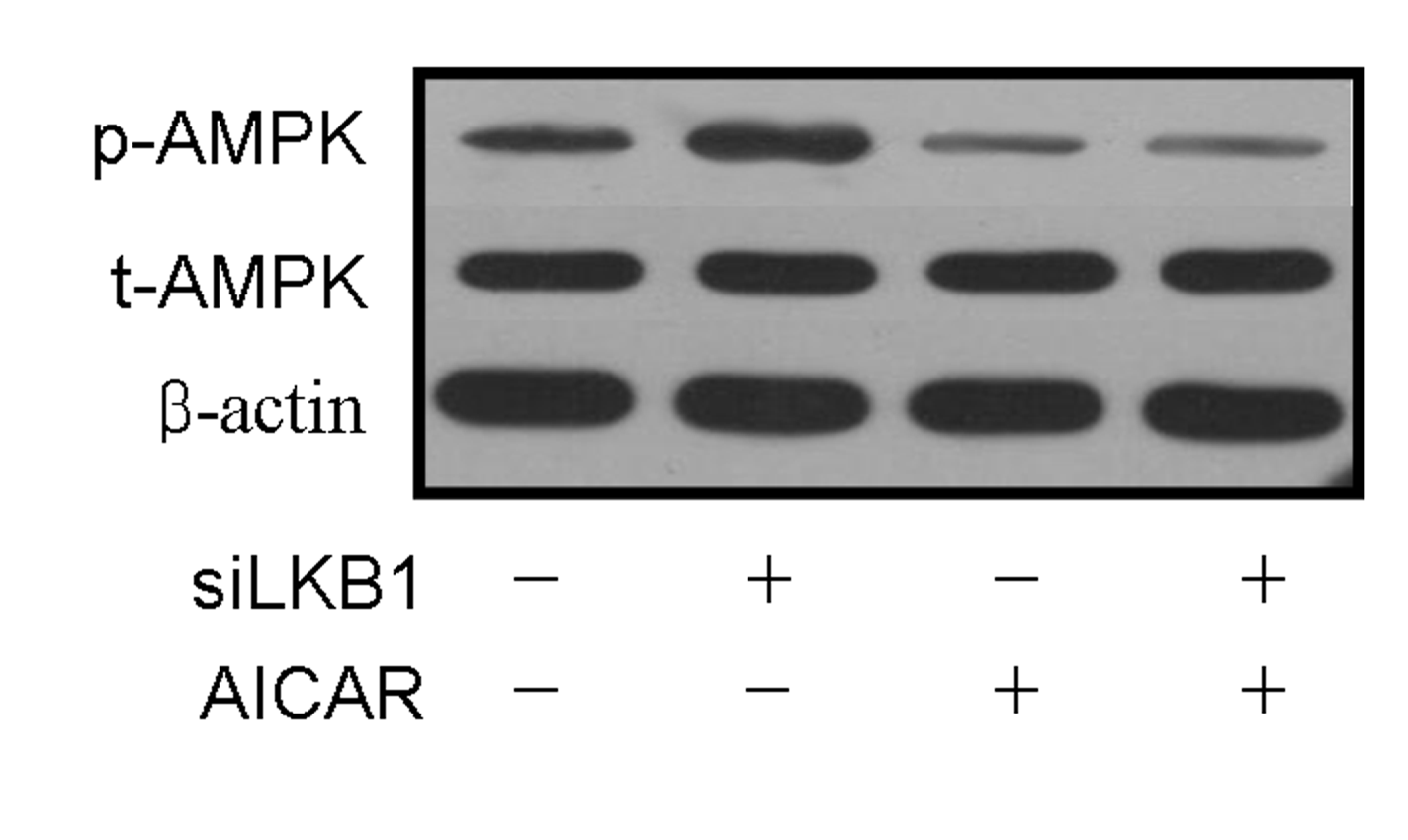

Supplement: Figure S3 — LKB1 knockdown inhibits AICAR-induced AMPK phosphorylation. LKB1 was transiently knockdown by specific siRNAs. AICAR (1 mM) was added into the medium at 22 hrs after transfection and protein was harvested for western blotting after another 1 hr. [file jcmm0018-0293-sd3.tif]

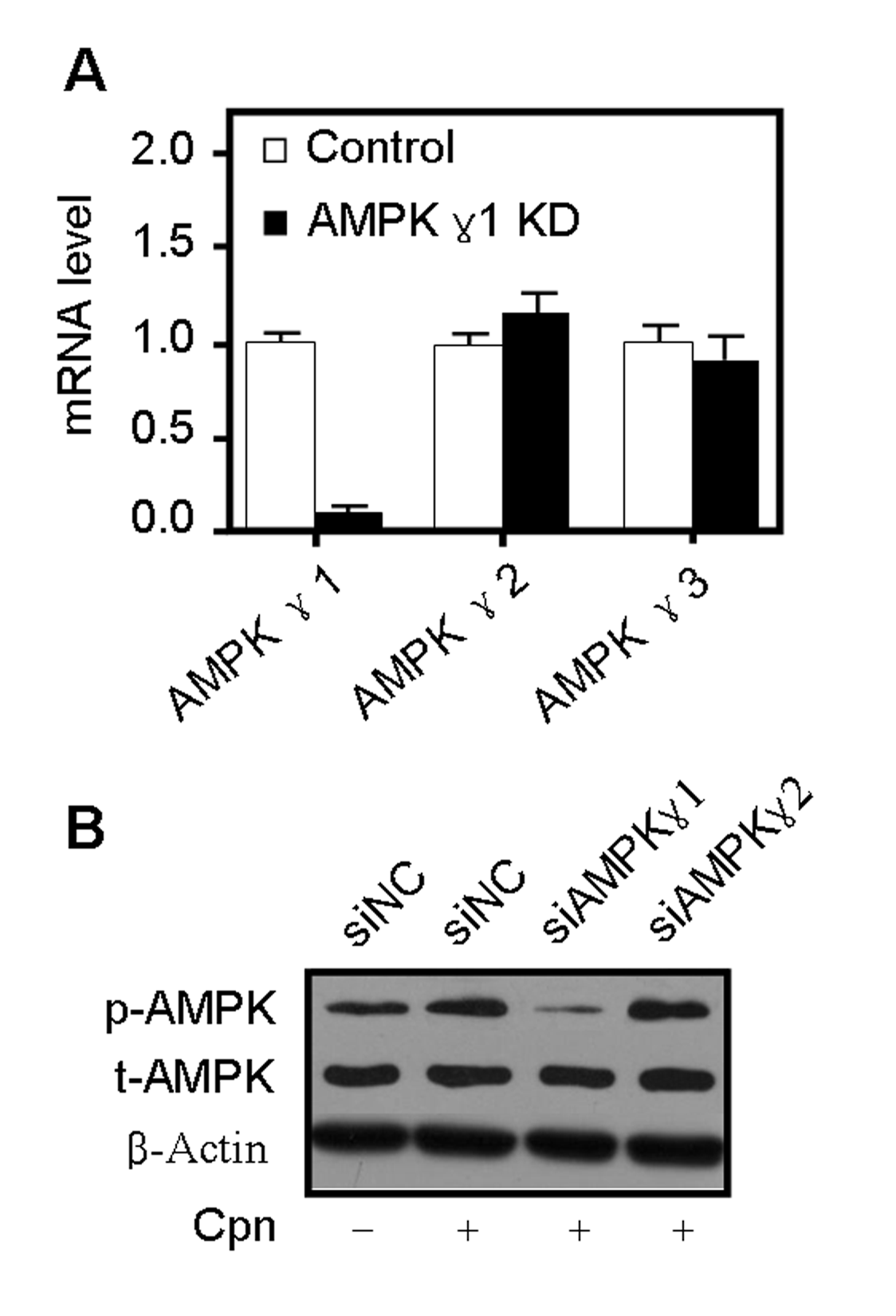

Supplement: Figure S4 — Effect of AMPKγ2 knockdown on cordycepin-induced AMPK phosphorylation. (A) AMPKγ1 knockdown does not affect the expression of other two isoforms of AMPKγ subunit as determined by real-time quantitative PCR. (B) Knockdown of AMPKγ2 does not influence cordycepin-mediated AMPK phosphorylation. Knockdown efficiency was assessed by real-time PCR. cpn: cordycepin. [file jcmm0018-0293-sd4.tif]

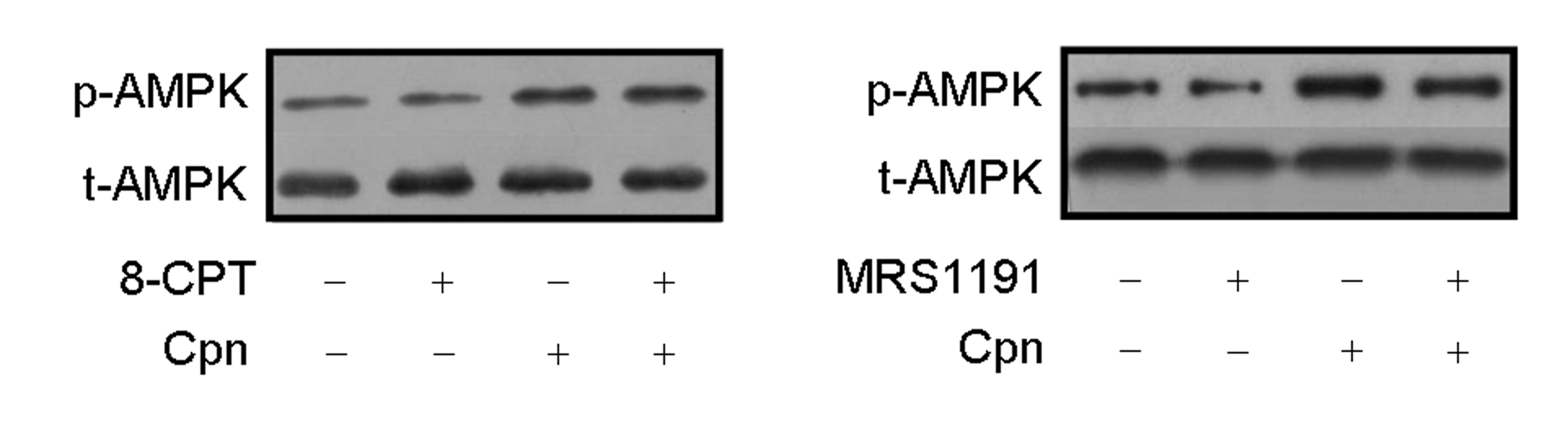

Supplement: Figure S5 — Inhibitors of adenine receptors A1 (8-CPT, 1 μM) (A) and A3 (MRS1191, 1 μM) (B) do not impair cordycepin (10 μM) -elicited AMPK phosphorylation. Before western blot, cells were treated with indicated reagents for 1 hr. cpn: cordycepin. cpn: cordycepin. [file jcmm0018-0293-sd5.tif]
