# Supplementary material for: Cordycepin activates AMP-activated protein kinase (AMPK) via interaction with the γ1 subunit
Source: J Cell Mol Med. 2013 Nov 28;18(2):293–304. doi: 10.1111/jcmm.12187 (PMC3930416; doi:10.1111/jcmm.12187)
Supplement: Table S1 — Oligonucleotide primers used in this study. [file jcmm0018-0293-sd6.doc]

Table S1. Oligonucleotide primers used in this work

| Primers | Sequence (5’-3’) |
| --- | --- |
| homo-LDLR-F | caatgtctcaccaagctctg |
| homo-LDLR-R | tctgtctcgaggggtagctg |
| homo-ACC-F | TGATGTCAATCTCCCCGCAGC |
| homo-ACC-R | TTGCTTCTTCTCTGTTTTCTCCCC |
| homo-ACOX1-F | GGGCATGGCTATTCTCATTGC |
| homo-ACOX1-R | CGAACAAGGTCAACAGAAGTTAGGTTC |
| homo-CPT1-F | cgtcttttgggatccacgatt |
| homo-CPT1-R | tgtgctggatggtgtctgtctc |
| homo-HMGR-F | ggacccctttgcttagatgaaa |
| homo-HMGR-R | ccaccaagacctattgctctg |
| homo-ApoB-F | TCGCCTGCCAAACTGCTTC |
| homo-ApoB-R | CATTGGTGCCTGTGTTCCATTC |
| homo-ABCA1-F | TGTCCAGTCCAGTAATGGTTCTGT |
| homo-ABCA1-R | AAGCGAGATATGGTCCGGATT |
| homo-ABCG1-F | GCCTACTGCAGACTCGTGTA |
| homo-ABCG1-R | GTCGAAGCTGACGAAGAACC |
| homo-LPL-F | ACAGAATTACTGGCCTCGATCC |
| homo-LPL-R | CTGCATCATCAGGAGAAAGACG |
| homo-PPARa-F | aaaagcctaaggaaaccgttctg |
| homo-PPARa-R | tatcgtccgggtggttgct |
| homo-PPARy-F | CATGGCAATTGAATGTCGTGTC |
| homo-PPARy-R | CCGGAAGAAACCCTTGCAT |
| homo-SREBP-1a -F | tgctgaccgacatcgaagac |
| homo-SREBP-1a -R | ccagcatagggtgggtcaa |
| homo-SREBP-1c-F | ccatggatgcactttcgaa |
| homo-SREBP-1c-R | ccagcatagggtgggtcaa |
| homo-SREBP-2-F | ctgcaacaacagacggtaatga |
| homo-SREBP-2-R | ccattggccgtttgtgtcag |
| homo-LXRa-F | tcgagtcacgccttggc |
| homo-LXRa-R | ggtagctgtttagcaaagtcaa |
| homo-FAS-F | CGGTACGCGACGGCTGCCTG |
| homo-FAS-R | GCTGCTCCACGAACTCAAACACCG |
| homo-UCP1-F | CTTGGTGTCGGCTCTTATCG |
| homo-UCP1-R | CCGTTGGTCCTTCGTTAGTG |
| homo-UCP2-F | GGTGGTCGGAGATACCAAAG |
| homo-UCP2-R | CTCGGGCAATGGTCTTG |
| homo-UCP3-F | GGGATTCTGGTCTTCACTGC |
| homo-UCP3-R | TCCAACCTTCCATTTTGTCC |
| homo-apoA1-F | GCAGAGACTATGTGTCCCAGTTG |
| homo-apoA1-R | CCAGTTGTCAAGGAGCTTTAGGTT |
| homo-apoB-F | Acgtattcttgtggtcatatgttgttt |
| homo-apoB-R | tctgtggttgacctgctttcc |
| homo-CYP7A1-F | GCATCATAGCTCTTTACCCAC |
| homo-CYP7A1-R | GGTGTTCTGCAGCAGTCCTGTAAT |
| homo-DGAT1-F | ccggcgtgggcagcg |
| homo-DGAT1-R | gcatcaccacacaccagttcagga |
| homo-DGAT2-F | gcgagcgtcaggccgagg |
| homo-DGAT2-R | gagggcggagaggatgctggat |
| homo-ACTIN-F | CCTGGCACCCAGCACAAT |
| homo-ACTIN-R | GCCGATCCACACACGGAGTACT |
